# The power of modelling pulsatile profiles

**DOI:** 10.1007/s10928-021-09743-2

**Published:** 2021-03-03

**Authors:** Michiel J. van Esdonk, Jasper Stevens

**Affiliations:** 1 Centre for Human Drug Research, Leiden, The Netherlands; 2 Department of Clinical Pharmacy and Pharmacology, University Medical Center Groningen, University of Groningen, Groningen, The Netherlands

**Keywords:** Statistical power, Deconvolution, Chronopharmacometrics, Population models, Endocrinology

## Abstract

The quantitative description of individual observations in non-linear mixed effects models over time is complicated when the studied biomarker has a pulsatile release (e.g. insulin, growth hormone, luteinizing hormone). Unfortunately, standard non-linear mixed effects population pharmacodynamic models such as turnover and precursor response models (with or without a cosinor component) are unable to quantify these complex secretion profiles over time. In this study, the statistical power of standard statistical methodology such as 6 post-dose measurements or the area under the curve from 0 to 12 h post-dose on simulated dense concentration–time profiles of growth hormone was compared to a deconvolution-analysis-informed modelling approach in different simulated scenarios. The statistical power of the deconvolution-analysis-informed approach was determined with a Monte-Carlo Mapped Power analysis. Due to the high level of intra- and inter-individual variability in growth hormone concentrations over time, regardless of the simulated effect size, only the deconvolution-analysis informed approach reached a statistical power of more than 80% with a sample size of less than 200 subjects per cohort. Furthermore, the use of this deconvolution-analysis-informed modelling approach improved the description of the observations on an individual level and enabled the quantification of a drug effect to be used for subsequent clinical trial simulations.

## Introduction

Drugs are administered with the aim to affect various physiological, biochemical and behavioral clinical markers to the benefit of patients. Many of these markers vary based on time-of-day and it is well recognized that the endogenous “circadian” clock lies at the basis of these often wave-shaped pharmacodynamic patterns. It is only logical to assume that optimizing the time of drug administration may increase clinical efficacy and safety in some cases, which indeed has been shown, e.g., for glucocorticoids, antihypertensives and antibiotics [1–4].

**Fig. 1:**
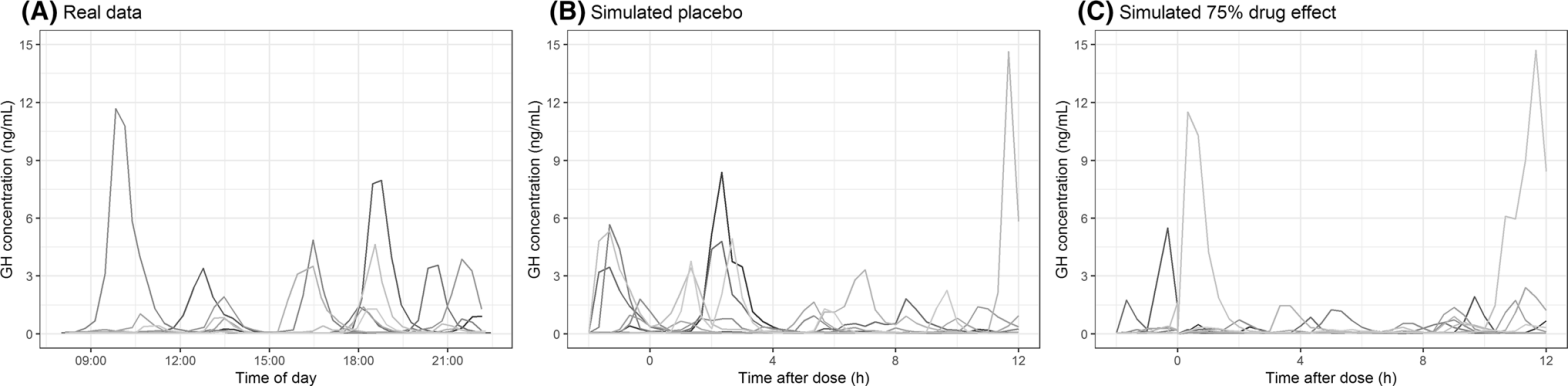
**a** Growth hormone profiles sampled at 20-min intervals during daytime of eight healthy male subjects. **b** Ten random simulated placebo growth hormone profiles simulated at 20-min intervals. **c** Ten random simulated growth hormone profiles with an inhibitory drug (t_max_ 2 h, t_1/2_ 3 h, 75% inhibition of pulsatile secretion at t_max_) administered at time 0. In-house data (**a**) and simulation model from Van Esdonk et al*.* [14]

In order to improve treatment of diseases that have clinically relevant circadian components, there is a growing interest to apply systems chronotherapeutics approaches [5]. Using mathematical models, wave shaped patterns can be described by single or double cosine functions over time-of-day, like e.g. heart rate, QT interval and blood pressure [6, 7]. Also, more complex biological processes that are under circadian control can be readily described with precursor-dependent indirect response (pool) models, with or without agonist–antagonist interaction submodels e.g. prolactin release by the pituitary [8, 9]. However, endogenous compounds that have a very short half-life and are released in pulses do not show such a nicely flowing curvature, as can be seen for endogenous growth hormone secretion presented in Fig. 1a, and are therefore less easy to capture using cosine functions or pool models. This release pattern is commonly encountered in endocrine systems (e.g. growth hormone, glucose, luteinizing hormone, cortisol) which creates a challenge for the correct description of such a profile in a modelling approach.

Due to the high level of intra- and inter-individual variability that exists on the pulsatile release of growth hormone (Fig. 1a), a combination of more than 2 or 3 cosinor functions would need to be adapted on an individual level for each individual profile and structural modifications to the model would be required on a day-to-day level to describe the data. This multitude of required parameters complicates the quantification of the individual endogenous release and might not result in accurate individual descriptions of the data [10]. Moreover, it complicates the quantification of a concentration-effect relationship and thus increases the required number of subjects in studies in order achieve adequate statistical power. Finally, simulations with variability on these parameters may result in non-realistic secretion shapes over time due to the interaction of multiple cosinor functions.

In order to quantitate the pulsatile release of growth hormone over time, a non-linear mixed effects (population) model can be used more accurately and more efficiently if the underlying pulse times are known, thereby reducing the number of parameters that need to be estimated by the model. In order for the identification of the underlying pulse times, we can make use of an initial deconvolution step on the dataset. In short, with deconvolution, the pulsatile pattern is analyzed on an individual level and the underlying pulse times are extracted [11, 12]. This deconvolution methodology works by randomly inserting pulses as underlying secretion variables and judging if inclusion of that pulse time improves the description of the data. The algorithm continues until the best combination of pulse times has been identified for an individual profile. The advantage of this method is that it can handle highly stochastic profiles in which the timing of the underlying stimulatory event is unknown.

Having quantified the pulse times a priori reduces the degrees of freedom in the model and enables the quantification of only the remaining parameters required to fit a pulsatile profile, such as the pulse amplitudes, pulse secretion width, and a continuous zero-order (non-pulsatile) endogenous release. Previous publications have shown that this methodology provides an accurate description of the pulsatile data and is capable of quantifying drug effects on highly variable datasets [13, 14]. Furthermore, this deconvolution-analysis-informed modelling approach can be used for more realistic clinical trial simulations that mimic the endogenous secretion pattern, in which the high variability from different sources (differences in pulse times, variability in pulse amplitude, variability in the pharmacokinetics, etc.) is incorporated.

Even though approaches exist for the quantification of densely sampled pulsatile concentration–time profiles, growth hormone concentrations are commonly studied by measuring only a single sample, or the mean of multiple samples as primary outcome [15]. This approach seems most likely taken for clinical feasibility and reduction of patient burden that would accompany obtaining a multi-hour dense profile. Unfortunately, even though less samples are required, these methods of statistical analysis oversimplify the complex secretory profile which may impact the statistical power when investigating significant changes between groups.

In this study we investigate the impact of several standard statistical methods and the potential advantage of applying a deconvolution-analysis-informed model on growth hormone data in a simulated clinical trial. The statistical power of quantifying a significant change between simulated placebo and active treated subjects was investigated by use of a (1) linear mixed effects model on 1 pre-dose growth hormone and 6 post-dose growth hormone measurements, (2) a linear model on the area under the growth hormone concentration curve on dense data sampled from 0 to 12 h (AUC_0-12 h_) post-dose, and (3) a deconvolution-analysis-informed modelling approach on dense growth hormone data sampled from 2 h pre-dose to 12 h post-dose, while simulating multiple inhibitory drug effect sizes (25%, 50%, and 75%) on the pulsatile secretion.

## Methods

### Simulation

A previously developed growth hormone model in healthy male volunteers was used for the simulation of growth hormone profiles [14]. This growth hormone model was based on dense concentration–time profiles with sampling performed at 20 min intervals for a duration of 14 h. The model was able to describe the individual pulsatile release of growth hormone secretion and each individual growth hormone pulse was quantified using a pulse secretion width, a pulse amplitude, and the time of the pulse parameter (Eq. 2). The pulse times were simulated for this study based on the mean and standard deviation of the interval in minutes between the pulses on log transformed data from this literature population (natural log mean = 4.33, natural log standard deviation = 0.98), resulting in realistic simulated profiles to be used for the analysis in this study (Fig. 1b and c). Based on the used parameters, an individual growth hormone concentration–time profile showed a mean of 7 pulses (range 0–15) during a 14 h timeframe and was highly variable across individuals. The simulated pulse times were included in the dataset as separate columns to be used in NONMEM V7.3 for simulation and parameter estimation [13].

The linear models and the deconvolution-analysis-informed analysis was performed on simulated data of a parallel study design with a sample size of 200 placebo and 200 active treated subjects. Active treated subjects received a hypothetical oral drug following 1-compartment distribution kinetics with first-order absorption and elimination (parameters fixed to: absorption rate constant = 0.95/h, volume of distribution = 65L, clearance = 15 L/h, dose = 102) resulting in a pharmacokinetic (PK) profile with a t_max_ at 2 h, a C_max_ of 1, and a half-life of 3 h. At the maximal concentration, the drug induced an inhibition of 25%, 50%, or 75% of the pulsatile secretion of growth hormone, which was implemented as a linear concentration-effect relationship (Eq. 1–4).

1 $$Effect \left(t\right)=Concentration(t)*slope$$

2 $${S}_{n}\left(\mathrm{t}\right)={\mathrm{e}}^{\mathrm{ln}\left({\mathrm{Amplitude}}_{n}\right)- \frac{1}{2} \cdot ({\frac{\mathrm{t}-{\mathrm{PulseTime}}_{n}}{\mathrm{SecretionSD}})}^{2}}*(1-Effect\left(t\right) )$$

3 $${GH}_{secretion}\left(\mathrm{t}\right)=baseline+{S}_{1}\left(t\right)+{S}_{2}\left(t\right)+{S}_{3}\left(t\right)\dots {S}_{n}(t)$$

4 $$\frac{d{C}_{GH}}{dt} = {GH}_{secretion}\left(\mathrm{t}\right)-{k}_{el}*{C}_{GH}(t)$$

where *slope* was fixed to 0.25, 0.5, or 0.75 to simulate the different effect sizes driven by the *Concentration* of the drug over time. In Eq. 2 and 3, *S*_*n*_*(t)* represents the secretion of pulse *n* at time *t*, *Amplitude*_*n*_ is the amplitude of pulse *n* and changes between each pulse within an individual, *PulseTime*_*n*_ is the time of maximum secretion for pulse *n*, extracted from the dataset and based on the initial deconvolution step, and *SecretionSD* is the standard deviation of the pulse width which is identical for all pulses in an individual. The *Amplitude* of a pulse changes between individual pulses based on the variance identified previously [14]. *Effect* is a proportional inhibition of the secretion of pulse *n* over time. The total growth hormone secretion at time t (Eq. 3) is a zero-order endogenous *baseline* secretion of growth hormone plus the sum of *S*_*n*_ for all pulses at time *t*, which is the input of the ordinary differential equation of the measured growth hormone concentration (*C*_*GH*_, Eq. 4). Growth hormone gets eliminated with the first-order elimination rate constant *k*_*el*_ and the differential equation is initialized at *baseline*.

This parameter set simulates a maximal inhibition of the pulsatile growth hormone secretion at 2 h post-dose with thereafter a decline of more than 3 half-lifes in a 12 h post-dose timeframe. No drug effect on the endogenous (non-pulsatile) *baseline* growth hormone secretion was implemented. Inter-individual variability on the *baseline*, *SecretionSD* and the *k*_*el*_ parameters were implemented based on the values of Van Esdonk et al*.*[14]. No inter-individual variability on the PK or on the PK/PD relationship was introduced in the simulation.

### Statistical analysis

Three statistical analysis methods on which the power was based were investigated: (1) a linear mixed effects model on 1 pre-dose growth hormone and 6 post-dose growth hormone measurements up to 12 h post-dose, (2) a linear model on the derived AUC_0-12 h_ based on growth hormone concentrations sampled every 20 min for 12 h post-dose, and (3) a deconvolution-analysis-informed modelling approach based on growth hormone concentrations sampled every 20 min starting 2 h pre-dose up to 12 h post-dose.

The 95% confidence interval of the estimated power for each methodology was calculated by 1000 iterations of the simulation and power estimation step of the 6 post-dose measurements and AUC_0-12 h_. The mean, minimal, and maximal power of 10 Monte-Carlo Mapped Power analyses for each effect size were plotted due to a lower degree of uncertainty in this outcome combined with computational limitations (2 threads, Intel® Xeon® CPU E5-2690 v2 @ 3.00gHz, MCMP runtime ~ 15 h per analysis).

#### Method 1: linear mixed effects on multiple post-dose samples

In the linear mixed effects model, 1 pre-dose growth hormone concentration was measured and growth hormone concentrations were determined at 1 h, 2 h, 4 h, 6 h, 8 h and 12 h post-dose, mimicking a sampling schedule of an early phase clinical trial study design. A linear mixed effects model with treatment by time and the pre-dose growth hormone concentration as fixed effects and subject identifier as random effects on the intercept was fitted to the data. The treatment contrast (active vs. placebo) was computed using the least-squares means and was transformed to the Cohen’s effect size. The power was determined based on the Cohen’s effect size with a two-sided two-sample t-test and an alpha of 0.05. The R syntax used for the linear mixed effects model is shown below:

$$lmer(GH\,concentration \sim Treatment\,*\,Sampling\,time\, + \,'pre - dose\,baseline\,concentration' \, + \,(1|Subject\,identifier), \, data\, = \, \ldots)$$

where *GH concentration* are the observed growth hormone concentrations, *Treatment* is a factor with two levels (active vs. placebo), *Sampling time* is a factor with the 6 levels of the sampling times, *pre-dose baseline concentration* is the pre-dose baseline growth hormone concentration for each individual and *Subject identifier* is a unique identifier for each individual.

#### Method 2: linear model on the area under the concentration–time curve up to 12 h post-dose

For the linear model, the AUC_0-12 h_ was calculated using the linear trapezoid rule on growth hormone concentrations from dosing up to 12 h post-dose, measured at 20 min intervals, and was analyzed with a linear model with treatment and the pre-dose growth hormone concentration as fixed effects. As only one observation was present per subject, no random effects were added to the model. The treatment contrast (active vs. placebo) was computed using the least-squares means and transformed to the Cohen’s effect size. The power was determined based on the Cohen’s effect size with a two-sided two-sample t-test and an alpha of 0.05. The R syntax used for the linear model is shown below:

$$lm(AUC_{0 - 12h} \sim Treatment\, + \,'{\text{pre - dose baseline concentration',}}\,{\text{data}}\,{ = } \ldots )$$

#### Method 3: deconvolution-analysis-informed modelling approach

The deconvolution-analysis-informed modelling approach was performed on growth hormone concentrations from 2 h pre-dose up to 12 h post-dose, sampled at 20-min intervals. A Monte-Carlo Mapped Power (MCMP) analysis was used as a tool to evaluate the power of this modelling approach on the quantification of a significant drug effect [16].

The MCMP analysis is a tool to quantify the statistical power of identifying a certain covariate or one or multiple parameters in a non-linear mixed effects (population) model. The MCMP uses the objective function value (OFV) on an individual level (iOFV) to compare the improvement in model fit in a model without the parameter present (reduced model) versus the model fit in a model with the parameter present (full model). In this case, the reduced model did not include the slope parameter, and therewith excluded the estimation of a PK/PD relationship as if no drug effect was present, whereas the full model did include this parameter.

In the first step of the MCMP analysis, the full model was used to simulate the growth hormone profiles over time for all individuals, including the drug effect for subjects that were randomized to receive the active treatment. Then, both full and reduced models were fitted to this dataset and the model fit on both models were compared to calculate the difference in the fit of the data on an individual level (ΔiOFV). Then, the sum of the ΔiOFV (Σ ΔiOFV) of a range of sample sizes (up to 200 subjects per treatment in a 1:1 ratio) were extracted at random and repeated for 10,000 iterations. For each sample size, the proportion of the iterations that shows a significant (p < 0.05) improvement in model fit, based on a ΣΔiOFV greater than 3.84 with 1 degree of freedom, was taken as the power for that explored sample size. This simulation, estimation, and sampling procedure was executed 10 times for each effect size to account for the level of variability that was present in simulated growth hormone concentrations. Only runs with successful minimization on both the full and reduced models were included in the analysis. No parameters were fixed in the full or in the reduced models.

### Software

All data transformations and visualizations were performed in R (V3.6.1) [17]. Power calculations of the linear (mixed effects) models were performed with the *pwr* package. Simulations and MCMP analysis were performed in NONMEM (V7.3) in conjunction with PsN (V4.8.1) [16, 18, 19].

## Results

**Fig. 2:**
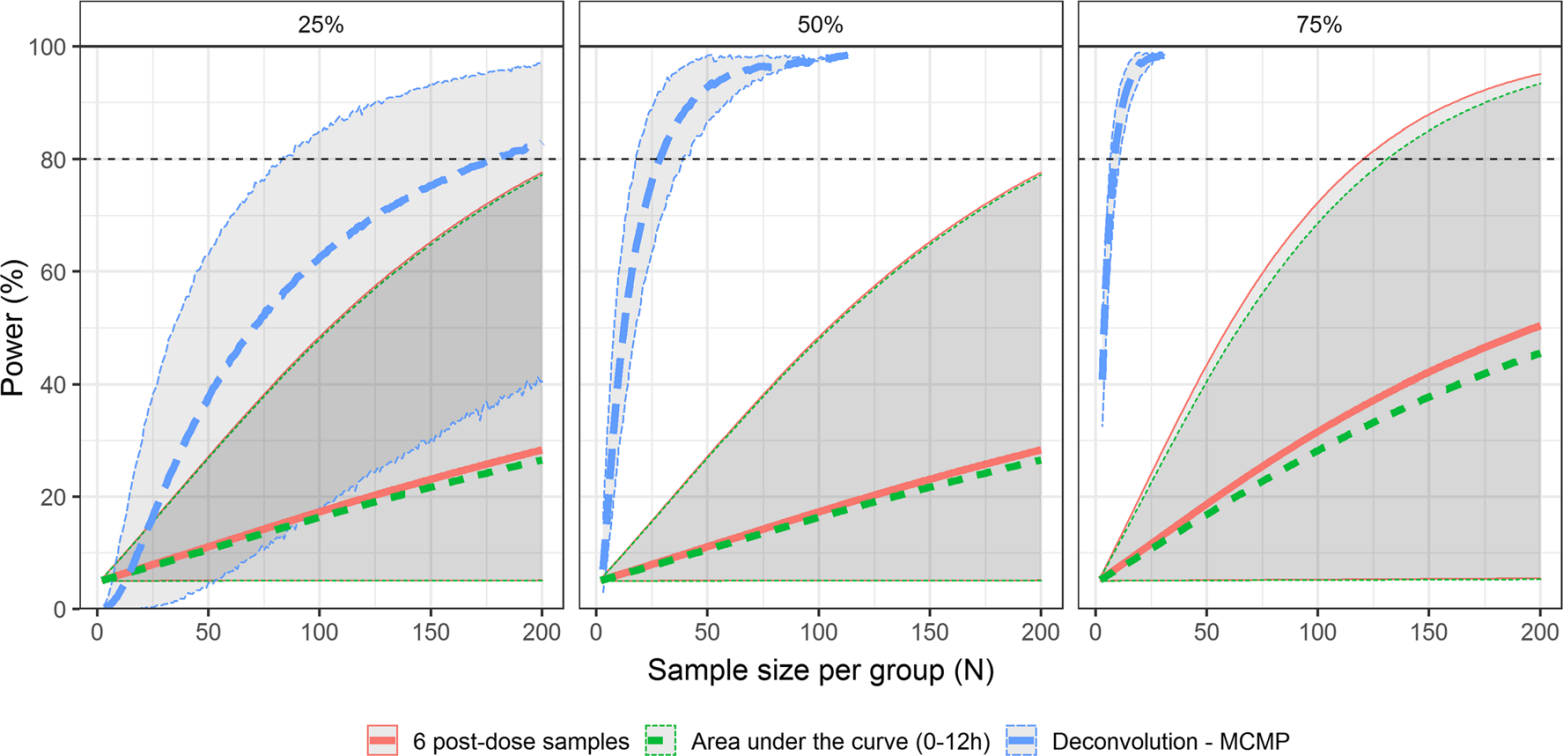
Mean power over sample size for the 6 post-dose measurements, the area under the curve from 0 to 12 h, and the deconvolution-analysis-informed model for three effect sizes (25%, 50%, and 75%). Effect sizes were implemented as the maximal effect reached at 2 h post-dose on the pulsatile secretion of growth hormone. Grey ribbon indicates 95% confidence interval. For the deconvolution-analysis-informed model, the grey ribbon indicates the minimal and maximal power of 10 Monte-Carlo Mapped Power repetitions

The estimated mean power at three effect sizes with three statistical analysis methods are presented in Fig. 2. The high variability in the simulated pulsatile profiles resulted in broad 95% confidence intervals when using the 6 post-dose measurements or the AUC_0-12 h_ outcomes in all scenarios, indicating that these analysis methods are sensitive to the highly variable nature of growth hormone secretion. These two statistical methods show a clear overlap, with low statistical powers in general, showing that, even though the AUC_0-12 h_ was based on a dense growth hormone concentration–time profile, 200 subjects per group did not reach an 80% power in all tested effect sizes. In comparison, the deconvolution-informed approach shows a significant increase in power with increasing sample size and shows that, especially when the drug effect is large, a low sample size (< 50 per group) can already result in a statistical power of more than 80%.

## Discussion

The choice of the study design and statistical method can have a large influence on the obtained statistical power when pulsatile biomarkers are being investigated. The use of a deconvolution-analysis-informed modelling approach on dense pulsatile concentration–time profiles improves the quantification of significant drug effects and increases the statistical power compared to a linear mixed effects model on limited samples or the AUC_0-12 h_ based on a highly similar dense sampling protocol. Due to the high levels of intra- and inter-individual variability in the secretion, the low endogenous baseline, in combination with the high and short bursts of growth hormone secretion, make standard statistical methodology lack statistical power.

The deconvolution-analysis-informed model provides an alternative approach to the use of cosinor functions for the modelling of pulsatile data and improves the quantification on an individual and population level. Clearly, sufficient data should be collected over time to apply the deconvolution technique to extract the individual pulse times. This requires an intensive sampling schedule, especially in the case of biomarkers with a short half-life like growth hormone, which may limit its clinical applicability and thus proof more applicable in a drug development setting. Furthermore, in all scenarios, the power calculations were based on a fixed population PK profile at a certain effect size. In reality, variability in the responsiveness of an individual to a drug and the variability in the PK parameters will further increase the level of variability in the growth hormone profiles over time, which could reduce the identified power in the current analysis and subsequent power analysis should therefore be performed while including additional information on the used treatment.

Even though profiles of other pulsatile hormones, or growth hormone secretion in acromegaly patients, will show a different pulsatile pattern, deconvolution may be of general use to better understand the characteristics of the underlying pulsatile data. Besides the increased power in estimating a significant drug effect, the deconvolution-analysis-informed model can be used for clinical trial simulations of new study designs and treatment regimens to better simulate realistic profiles which enhances the accuracy of model predictions for future studies.
